# Conversion Surgery in Gastric Cancer Carcinomatosis

**DOI:** 10.3389/fonc.2022.852559

**Published:** 2022-03-08

**Authors:** Paolo Morgagni, Leonardo Solaini, Luca Saragoni, Manlio Monti, Martina Valgiusti, Giovanni Vittimberga, Giovanni Luca Frassineti, Massimo Framarini, Giorgio Ercolani

**Affiliations:** ^1^ General and Oncologic Surgery, “Morgagni-Pierantoni” Hospital, Forlì, Italy; ^2^ Department of Medical and Surgical Sciences, University of Bologna, Bologna, Italy; ^3^ Pathology Unit, “Morgagni-Pierantoni” Hospital, Forlì, Italy; ^4^ Department of Medical Oncology, IRCCS Istituto Romagnolo per lo Studio dei Tumori (IRST) “DinoAmadori”, Meldola, Italy

**Keywords:** gastric cancer, conversion surgery, HIPEC, positive peritoneal cytology, metastatic disease

## Abstract

**Background:**

After the REGATTA trial, patients with stage IV gastric cancer could only benefit from chemotherapy (CHT). However, some of these patients may respond extraordinarily to palliative chemotherapy, converting their disease to a radically operable stage. We present a single centre experience in treating peritoneal carcinomatosis from gastric cancer.

**Methods:**

All patients with stage IV gastric cancer with peritoneal metastases as a single metastatic site operated at a single centre between 2005 and 2020 were included. Cases were grouped according to the treatment received.

**Results:**

A total of 118 patients were considered, 46 were submitted to palliative gastrectomy (11 were considered M1 because of an unsuspected positive peritoneal cytology), and 20 were submitted to Hyperthermic Intraperitoneal Chemotherapy (HIPEC) because of a <6 Peritoneal Cancer Index (PCI). The median overall survival (OS) after surgery plus HIPEC was 46.7 (95% CI 15.8–64.0). Surgery (without HIPEC) after CHT presented a median OS 14.4 (8.2–26.8) and after upfront surgery 14.7 (10.9–21.1). Patients treated with upfront surgery and considered M1 only because of a positive cytology, had a median OS of 29.2 (25.2–29.2). The OS of patients treated with surgery plus HIPEC were 60.4 months (9.2–60.4) in completely regressed cancer after chemotherapy and 31.2 (15.8–64.0) in those partially regressed (*p* = 0.742).

**Conclusions:**

Conversion surgery for peritoneal carcinomatosis from gastric cancer was associated with long survival and it should always be taken into consideration in this group of patients.

## Introduction

Peritoneal carcinomatosis is the most frequent metastatic site in gastric cancer (1). The findings from the REGATTA trial (2) indicate that patients with stage IV gastric cancer could only benefit from chemotherapy, regardless of the metastatic site; however, in other studies, these patients may respond extraordinarily to palliative chemotherapy, converting their disease to a radically operable stage (3) and showing promising results in a much selected group (3–8).

Considering the peritoneal metastatic site, a radical procedure associated with Hyperthermic Intraperitoneal Chemotherapy (HIPEC) seemed to be a valuable option to improve survival (6). However, the Japanese PHOENIX trial, which reported results comparing intraperitoneal/intravenous versus intravenous preoperative treatment failed to find significant differences between these procedures (9).

Interestingly, there is a different approach to HIPEC or conversion surgery by Eastern and Western authors; whereas Eastern authors presented studies where gastrectomy was proposed after chemotherapy and only if a second laparoscopy could confirm the absence of carcinomatosis, the Western ones considered HIPEC in cases which presented a minimal carcinomatosis [peritoneal cancer index (PCI) <6)] after palliative chemotherapy (10).

In this context, this study aims to present an Italian single center experience on patients with peritoneal carcinomatosis as the single metastatic site.

## Material and Methods

### Patient Recruitment

Between 2005 and 2020, 913 patients were operated on for gastric cancer at the “Morgagni-Pierantoni” General Hospital, in Forlì. Of these, 118 presented peritoneal metastases as a single metastatic site. These cases were all regularly discussed at multidisciplinary meetings during which different approaches were explored from the upfront surgery to conversion according to the guidelines in force at that time

These 118 patients were grouped according to the treatment received: a) patients submitted to upfront surgery and b) patients submitted to palliative chemotherapy and then surgically re-evaluated for resection alone or resection plus HIPEC (11). The peritoneal status was measured with the PCI. Patients with non-peritoneal distant metastases were excluded. Morbidity was classified in accordance with the Clavien–Dindo classification (12).

All surgically treated patients were then submitted to post-operative chemotherapy. Tumor stage was presented according to the Union for International Cancer Control (UICC)/American Joint Committee on Cancer (AJCC) (13).

All procedures performed in this study were in accordance with the ethical standard of the Area Vasta Romagna Ethics Committee (approval n° 5707/2020-I.5/264 on July 3, 2020), with the 1964 Declaration of Helsinki and its later amendments, and with the Good Clinical Practice (GCP) guidelines. Informed consent from patients was collected as instructed.

### Statistical Analysis

Continuous data was presented as the median and interquartile range (IQR). Fisher’s exact test was used to compare categorical variables which were presented as numbers and percentages. The Kaplan–Meier curve was used to calculate survival rates, and differences in survival rates between subgroups were assessed by the log-rank test. Overall survival was defined as the time between surgery and death or last follow-up. The median follow-up and IQR was found using the Kaplan–Meier function as suggested by Schemper and Smith (14). A 95% confidence interval (CI) was reported when required. Analyses were performed with MedCalc^®^ for Windows^®^ (version 10.2.0.0; MedCalc Software, Ostend, Belgium).

## Results

**Figure 1:**
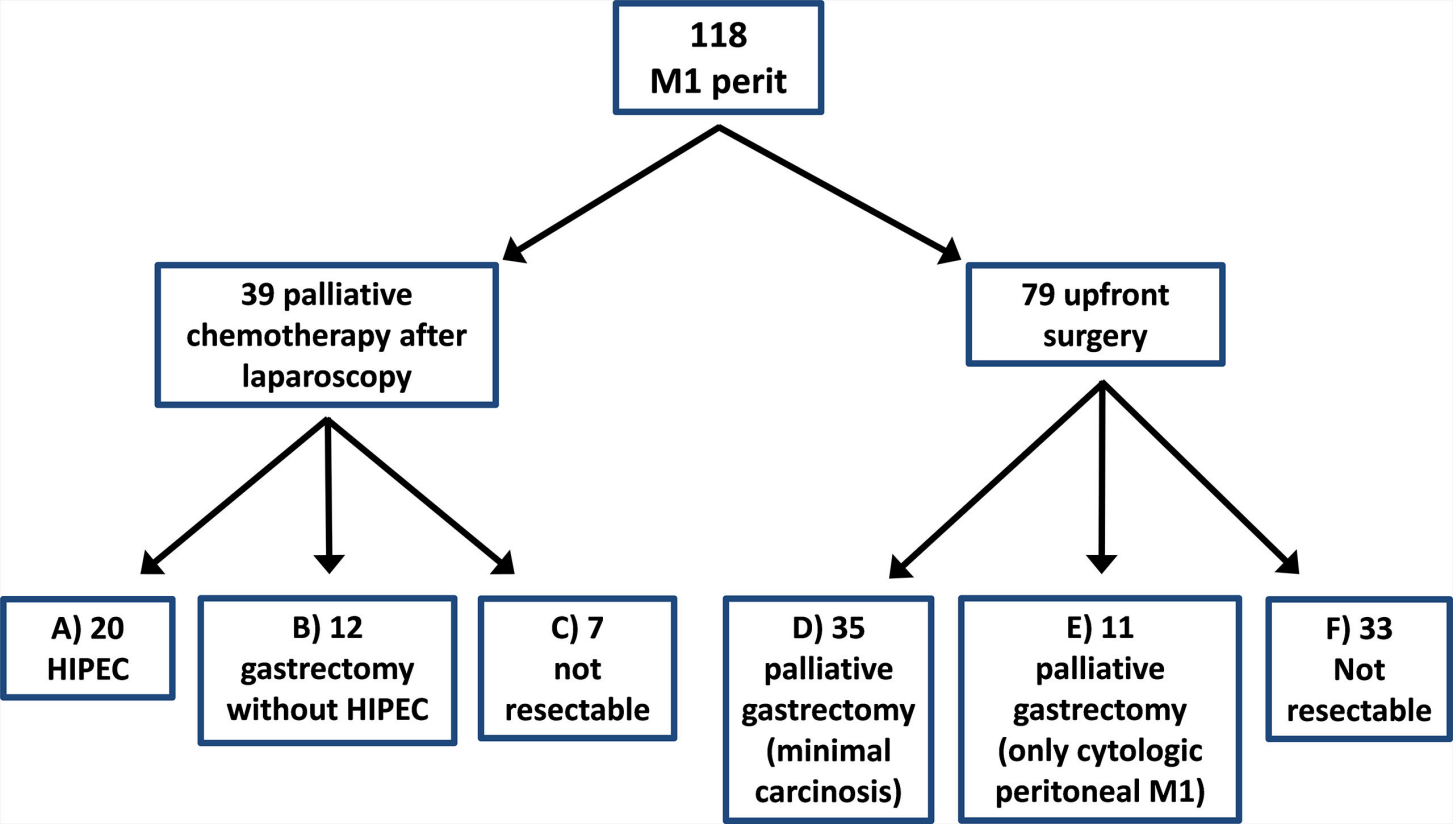
Flow-chart shows the types of treatment.

In total, 118 patients with peritoneal carcinomatosis as the exclusive metastatic site were included in the study. The type of treatment received is shown in the flow-chart, **Figure 1**. Clinicopathological characteristics and operative details are presented in **Table 1**.

**Table 1:** Patients’ characteristics.

Variables	Palliative CT+ HIPEC	CT+ surgery	Upfront surgery	Explorative laparotomy
	(No. = 20)	No. = 12	No. = 46	No. = 40
**Gender**				
Male	13	8	34	22
Female	7	4	12	18
**Age**	61.05 (range 29–78)	66.5 (range 53–77)	76.7 (range 61–90)	67.7 (range 37–89)
**T stage**				
cT2	0	1	–	–
cT3	2	0	–	–
cT4a	15	10	–	–
cT4b	3	1	–	–
yT0	3	1	0	0
yT1	3	0	0	0
yT2	1	0	0	0
yT3	4	4	10	0
yT4a	9	6	32	13
yT4b	0	1	4	27
**N stage**				
cN0	0	2	–	–
cN+	11	7	–	–
Nx	9	3	–	–
yN0	8	3	1	–
yN1	4	3	6	–
yN2	3	1	8	–
yN3a	2	3	14	–
yN3b	3	2	17	–
**Lauren histotype**				
Intestinal	14	5	26	20
Diffuse	3	6	15	20
Mixed	3	1	5	
**Site**				
cardias	5	–	9	7
fundus	3	1	6	4
corpus	4	4	8	11
antrum	6	5	18	10
all	2	2	5	10
**Regression (Becker)**				
1a	3	0	–	–
1b	4	3	–	–
2	3	1	–	–
3	10	8	–	–
**Gastrectomy**				
Subtotal	5	4	20	0
Total	15	8	26	0
Explorative	0	0	0	40
**Cytology**				
Pretreatment pos	20	2	–	–
Pretreatment neg	0	4	–	–
Pretreatment Not det	0	6	–	–
Surgery pos	10	4	16	18
Surgery neg	10	5	4	2
Surgery not det	0	3	26	20

Of the 79 patients treated with upfront surgery, 33 received only an exploratory laparoscopy because of diffuse carcinomatosis, and 46 had palliative gastrectomy. Eleven of these upfront surgery patients were considered M1 only because of a positive peritoneal cytology without macroscopic carcinomatosis (**Figure 1**).

Thirty-nine patients were submitted to an explorative approach and then proposed for oncologic treatment before re-evaluation. Induction chemotherapy included FOLFOX (5-fluorouracile, leucovorin ed oxaliplatin) in 12 cases (30.8%), PELF (Cisplatin, Epirubicin, Leukovorin, 5-Fluoruracil) in 12 (30.8%), FLOT (Fluorouracil, Leucovorin, Oxaliplatin and Docetaxel) in 11 (28.2%) and other different treatments in the remaining 4 cases (10.2%). At re-evaluation, twenty of these were found as partially (n = 10) or totally regressed (n = 10) and were submitted to surgery plus HIPEC; twelve patients were submitted to palliative surgery and seven could not be operated on because of progression during treatment.

Clavien–Dindo >2 complications rate was 20.0 (*n* = 4) in patients treated with HIPEC and 13 (37.1%) in patients who had upfront surgery (*p* = 0.234). Mortality was similar between the groups (1 death after pancreatitis and leakage vs. 2 deaths due to leakage/bleeding and perforation).

**Figure 2:**
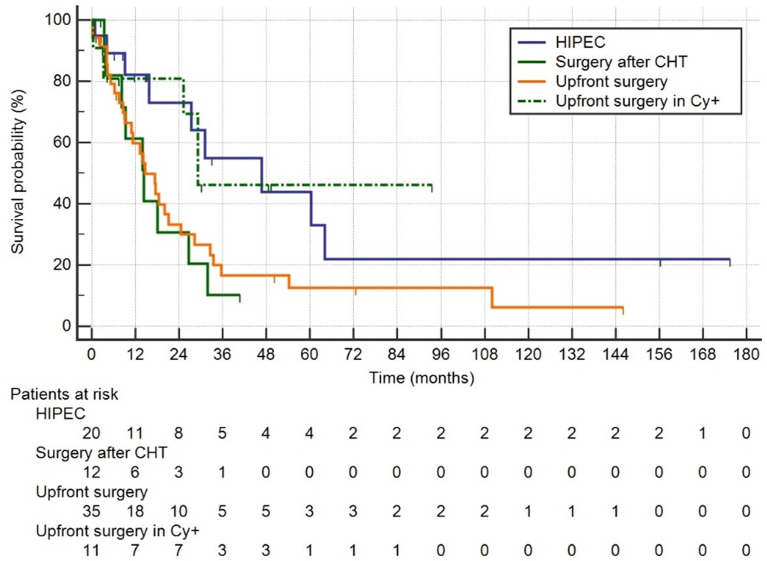
Group1: HIPEC, palliative chemotherapy followed by surgery plus HIPEC; Group 2: surgery after CTH without HIPEC; Group 3: upfront surgery; Group 4: upfront surgery in positive Cy+ only.

Kaplan–Meier curves are shown in **Figure 2**. Patients who had surgery plus HIPEC after chemotherapy had a median OS of 46.7 months (95% CI 15.8–64.0), those who had surgery after CHT 14.4 (8.2–26.8), those after upfront surgery 14.7 (10.9–21.1), and finally those who had upfront surgery for positive peritoneal cytology had a median survival of 29.2 months (14.7–29.2) (*p* = 0.050). Median follow-up was 50 months (IQR 15–110).

**Figure 3:**
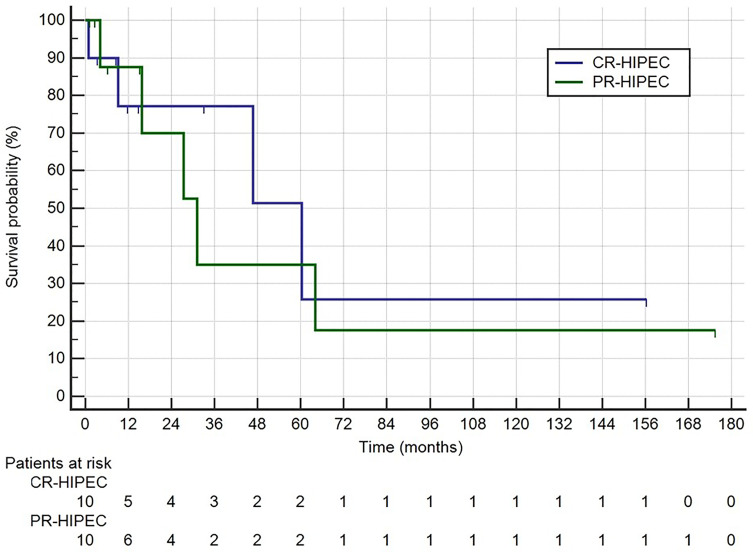
Survival rates for Group 1: CR-HIPEC, completely regressed followed by surgery plus HIPEC; Group 2: PR-HIPEC, partially regressed followed by surgery plus HIPEC.

Among the 20 patients who had R0 surgery plus HIPEC median survival was 60.4 (9.2–60.4) months in the group (*n* = 10) who had surgery plus HIPEC after a complete regression of peritoneal carcinomatosis (and peritoneal cytology) following chemotherapy (CR-HIPEC) versus 31.2 (15.8–64.0) in the patients (*n* = 10) who had a PCI <6 or positive peritoneal cytology after chemotherapy followed by surgery plus HIPEC (PR-HIPEC) (*p* = 0.742) (**Figure 3**).

## Discussion

The treatment of peritoneal carcinomatosis from gastric cancer represents the most challenging and intriguing treatment frontier and only a multidisciplinary approach may help in achieving the best results.

In 2016, Yoshida et al. classified all metastatic patients into four classes, differentiating macroscopic peritoneal involvement or not, and proposing conversion surgery especially for patients without peritoneal involvement (3). The worst results, nonetheless, sometimes gave positive outcomes, as observed by the same author in a selected group of patients with peritoneal involvement with a median survival time of 31.0 for category three (previously unresectable except for local palliation) and 24.7 for category four (previously non curable metastases) (15).

The Korean and Japanese REGATTA trial indicated only chemotherapy for stage IV, and this is currently proposed in Korean guidelines (16).

In Japan some patients can be considered for surgery after chemotherapy and some promising results have been reported with conversion surgery after S1 or intraperitoneal paclitaxel; Yasufuku et al. reported a three-year survival rate of 76.9% in positive cytology patients (4), and Ishigami et al. reported 30.5 months median survival time after chemotherapy and preoperative HIPEC (5).

Unfortunately, in the West, gastric cancer treatments without the S1 option were associated with the worst results. Peritoneum represents a sort of barrier for chemotherapy and its involvement cannot be approached using conventional treatments. Rau et al., showed a median survival of 18 months in patients treated with surgery plus HIPEC (6) and Passot et al. showed similar results (17).

This poor prognosis is generally due to late diagnosis; early stage carcinomatosis, which could give some hope for cure, is difficult to be diagnosed as staging laparoscopy, which can help in defining the peritoneal involvement, is rarely performed.

Moreover, peritoneal carcinomatosis can be associated with other hidden metastatic sites often not detected at diagnosis and these metastases cannot be cured with a local peritoneal treatment.

Intraperitoneal hyperthermic chemotherapy failed to show clear benefits in patients with massive involvement and its use has also been discussed for gastric cancer at early stages (10).

In the Japanese trial PHOENIX, HIPEC was preoperatively proposed as intraperitoneal weekly chemotherapy through a port associated with intravenous treatment. In the second arm of this trial, patients received only intravenous chemotherapy; results did not find any advantages for preoperative HIPEC.

Of the specific treatments proposed such as HIPEC, bidirectional, and PIPAC, these probably present some results only for a subset of patients; for the other patients there is sometimes only a lesser amount of ascites (9, 18).

Our experience collected patients over a long period of time and some of the approaches were changed; in the last ten years, we generally proposed HIPEC for a very selected group of patients with positive cytology or small carcinomatosis detected at laparoscopy and always performed before preoperative treatment.

All the patients were firstly submitted to systemic palliative chemotherapy, generally with FOLFOX treatment and this was usually performed by our oncologist for metastatic treatment. This approach, which postponed surgery for a median of 3 months, improved the selection of those patients with rapidly advancing cancer by treating them only with chemotherapy and avoiding unnecessary surgery.

Responder patients if not R0, but potentially radically resectable with a PCI <6, were submitted to surgical treatment with gastrectomy and HIPEC.

The most interesting result of our study was that survival rates after HIPEC did not significantly differ from patients submitted to surgery and completely regressed after palliative preoperative treatment and those patients operated on even if with only a few carcinomatosis, that is: if R0 could be reached, HIPEC was also proposed to patients with small areas of resectable carcinomatosis. These patients presented results as good as those completely regressed after preoperative chemotherapy.

This approach is completely different from the Asian experience which recommended surgery only to patients with complete regression and it also differs from some Western experiences, which presented extended indications for surgery plus HIPEC (10).

Another surprising result was the good survival rates of patients with positive cytology treated without HIPEC, but with upfront surgery. These patients presented promising outcomes with a median survival of 29.2 months (14.7–29.2).

Considering patients M1 only because of peritoneal positive cytology, good survival rates have been presented also by other authors: in 2019, Kim et al. proposed to classify positive cytology in a particular subset with massive lymphatic involvement (N3b) patients because of unlike other stage IV carcinosis; these patients present similar survival rates (19).

Even if we generally think that patients with advanced carcinomatosis may be better treated with oncologic treatment as proposed by the Korean REGATTA trial, we could, perhaps, select a subset of patients suitable for good surgical results by using a real multimodal approach.

Our study has a few limitations. First, it is a retrospective study and it carries the bias linked to its nature. Second, during the fifteen year-long study interval, several major changes in the management of stage IV gastric cancer have been introduced and this resulted in the heterogeneity of treatment seen in our analysis. As such, staging laparoscopy was not routinely performed as it was after 2013 (20, 21) and this may have had an impact on the type of treatment received. Finally, it must be observed that the upfront surgery group may have included a higher rate of symptomatic cases and/or elderly patients who were not fit for preoperative treatment. Those factors should be taken into account in the interpretation of the survival curves.

## Conclusion

Prognosis in peritoneal metastasis is generally poor; however, the good results observed in the HIPEC subset of patients, gives hope that it will be possible to select some patients fit for surgery and stimulate research in this direction.

## Data Availability Statement

The raw data supporting the conclusions of this article will be made available by the authors, without undue reservation.

## Ethics Statement

The studies involving human participants were reviewed and approved by the Area Vasta Romagna Ethics Committee (approval n° 5707/2020-I.5/264 on July 3rd, 2020). The patients/participants provided their written informed consent to participate in this study.

## Author Contributions

PM designed the study. LSo performed the statistical analysis. PM, LSo, and MM wrote the paper with input from all authors. All authors listed have made a substantial, direct, and intellectual contribution to the work and approved it for publication.

## Conflict of Interest

The Handling Editor declared a past co-authorship with the authors GE, LS and PM.

## Publisher’s Note

All claims expressed in this article are solely those of the authors and do not necessarily represent those of their affiliated organizations, or those of the publisher, the editors and the reviewers. Any product that may be evaluated in this article, or claim that may be made by its manufacturer, is not guaranteed or endorsed by the publisher.
